# Integrating the international medical graduate

**DOI:** 10.1016/j.fhj.2024.100221

**Published:** 2024-12-16

**Authors:** Julia McLaughlin, Katherine Baker, Tracy Sandell, Shirly Mathias, Subramaniam Nagasayi, Ilona Schmidt, Christopher James

**Affiliations:** Withybush General Hospital, Haverfordwest, Wales

## Abstract

**Introduction:**

The growing utilisation of international medical graduate (IMG) doctors in the UK has uncovered a need for hospitals to establish induction programmes that harness supportive environments for these doctors to learn within the specific context of the NHS. Currently, IMGs are under-supported at local and national levels.

**Method:**

The educational team within Withybush Hospital have designed and delivered a robust IMG induction programme. The aim was to improve IMG doctors’ clinical confidence within this specific setting. Feedback generated from the induction was provided to educational supervisors (ES) on their tutees. This induction was evaluated by questionnaires for IMGs following the induction activities, and annually for educational supervisors to assess their views.

**Outcome:**

Feedback was overwhelmingly positive with reported increased confidence post sessions. 100% of educational supervisors found the feedback provided to them very useful, allowing them to identify areas that the doctor needed support in, and aiding in the collaborative creation of development plans.

**Conclusion:**

NHS hospital-specific induction programmes for IMG doctors help to improve their clinical confidence and support their integration into their new role and environment. Additionally, educational supervisors value early, highly specific feedback generated from induction activities on the doctors they are supervising.

## Highlights

• International Medical Graduates need a robust, multifaceted induction to function as doctors in the NHS.
• Induction activities should be highly specific to the International Medical Graduate's new working environment.
• Induction needs to occur early in the onboarding process to ensure confidence is gained before working out-of-hours.
• International Medical Graduates benefit from the sharing of feedback generated from the induction with their educational supervisors.

## Introduction

Withybush Hospital is a small, district general hospital in Pembrokeshire, with holidaymakers increasing patient populations in the summer. The rural location makes retention and recruitment of doctors a challenge, resulting in the increasing need to recruit many international medical graduates (IMGs).^1^, ^2^ Replicated across the UK, with 44% of doctors currently on the GMC register having completed their primary medical qualification outside the UK.^2^^,^^3^ IMGs usually have limited experience of working in the NHS, and hospitals lack formal support networks for them.^4^

National research has shown that 52% of IMGs had no induction into the NHS, and 59% knew of another IMG who was referred to the GMC due to ‘lack of knowledge of NHS, bias, communication difficulties and cultural differences’.^4^

In a local survey of IMGs, doctors reported that they felt they had ‘no support’, and starting work was reported as ‘dark times’.

Considering the national and local research, it became apparent that Withybush Hospital needed to provide a tailored induction programme, to support and equip IMGs to work in the NHS, but also at Withybush specifically.^5^, ^6^, ^7^, ^8^, ^9^ This aimed to directly improve the doctors’ welcome into practice in the NHS and west Wales through a supportive induction, as well as indirectly ultimately protecting and aiding patient safety. Beyond an initial induction, measures were also needed to ensure supervisor engagement and opportunities for development.^6^^,^^7^^,^^9^^,^^10^

## Solution/methodology

As an educational team, we developed an IMG induction programme specific to our setting. From our clinical experience, IMG educational supervisor input, and piloting of the induction activities, four areas were identified. The induction was delivered on a monthly rolling roster to ensure that IMGs who were onboarding sporadically would be enrolled as early as possible.^7^ These sessions were regarded as crucial for IMGs to adapt to working within Withybush.^6^^,^^7^ Thus far, 37 IMG doctors have completed this evolving four-stage programme, as detailed below.

1. NHS professional skills
  *Designed as a pre-learning component, this session enabled IMGs to gain the tools to apply in the subsequent simulation sessions. This aimed to highlight the importance of documentation and effective handover to improve communication, continuity of care and patient outcomes, which incorporated local learning from events. This incorporated essential skills within the NHS, based on areas that were identified as unfamiliar in previous cohorts, such as documentation, on-call tips, medication charts and ABCDE assessment.*^6^^,^^9^
2. Clinical skills refresher
  *It was identified that clinical skills expectation, knowledge and application vary across healthcare settings and countries. This session allowed for familiarisation with local equipment and procedures, enhancement of existing skills, and highlighted the opportunities for further development.*
3. Emergency ABCDE simulation
  *This session covered up to four key emergency presentations and allowed for knowledge application within a simulated Withybush Hospital-specific environment. This also gave the opportunity for expectations of a UK doctor to be simulated for each individual, followed by a group debrief, to ensure whole group learning. The cases highlighted essential emergency protocols such as sepsis six, medical emergency calls, structured guideline bundles, and the major haemorrhage protocol.*
4. On-call ward cover simulation
  *This session facilitated an opportunity for doctors to assess, investigate, form a differential diagnosis, and manage acutely unwell cases. The focus of this simulation was to use local paperwork and guidelines to manage common on-call ward cover scenarios including a simulated handover to the night team. The aim was to enable these doctors to gain experience of specific tasks before facing such situations out of hours in the clinical environment. Ensuring the simulations were as locally realistic as possible, the cases also integrated Welsh names and addresses.*^9^*Written feedback was generated from observation of this simulation, which was shared with the IMG doctor and their educational supervisor.*^10^*This feedback provided the foundation for professional development plans to be discussed during supervisor meetings.*^10^

The support continued with signposting to relevant GMC/NHS induction resources, links to local training opportunities, as well as access to an e-portfolio. Mentoring was offered by a consultant within 3 months of onboarding, and an ARCP-style review at the end of the IMG’s first year provided an opportunity for development in a structured and supportive manner. IMGs are also offered entry into a buddy system to link them with IMG colleagues within the hospital.

Feedback from participant questionnaires informed further development of the programme.

An annual survey of ESs was also conducted to quantify the usefulness of the written feedback provided to them.

## Outcome

Questionnaire responses were collected over a 13-month period, and response rates varied for different groups of IMGs who undertook the induction. Where data are presented, the number of participants is the aggregated total of all respondents for that survey over the period of the study. The low initial self-reported confidence ratings in many of the interventions further confirm the need for this induction programme.

### Professional skills

100% (n=12) of participants strongly agreed that the NHS professional skills session was useful and relevant. The majority (7/12) regarded the review of the ABCDE assessment as the most helpful topic. They reported that the session was ‘interactive and pragmatic’ and found it ‘good to touch the base, especially as an IMG, to know how things work, what's our role, what's expected of us etc’. They understood the relevance of the foundational knowledge to work in the NHS reporting; ‘I gained new knowledge about DAL* and post-take rounds. This was really important’ (*DAL - discharge advice letter).

### Emergency ABCDE simulation session

Eleven participants completed the survey following the emergency ABCDE simulation, and 64% (n=7) of participants surveyed reported that their key learning was the importance of using a structure when completing an ABCDE assessment.

**Fig. 1:**
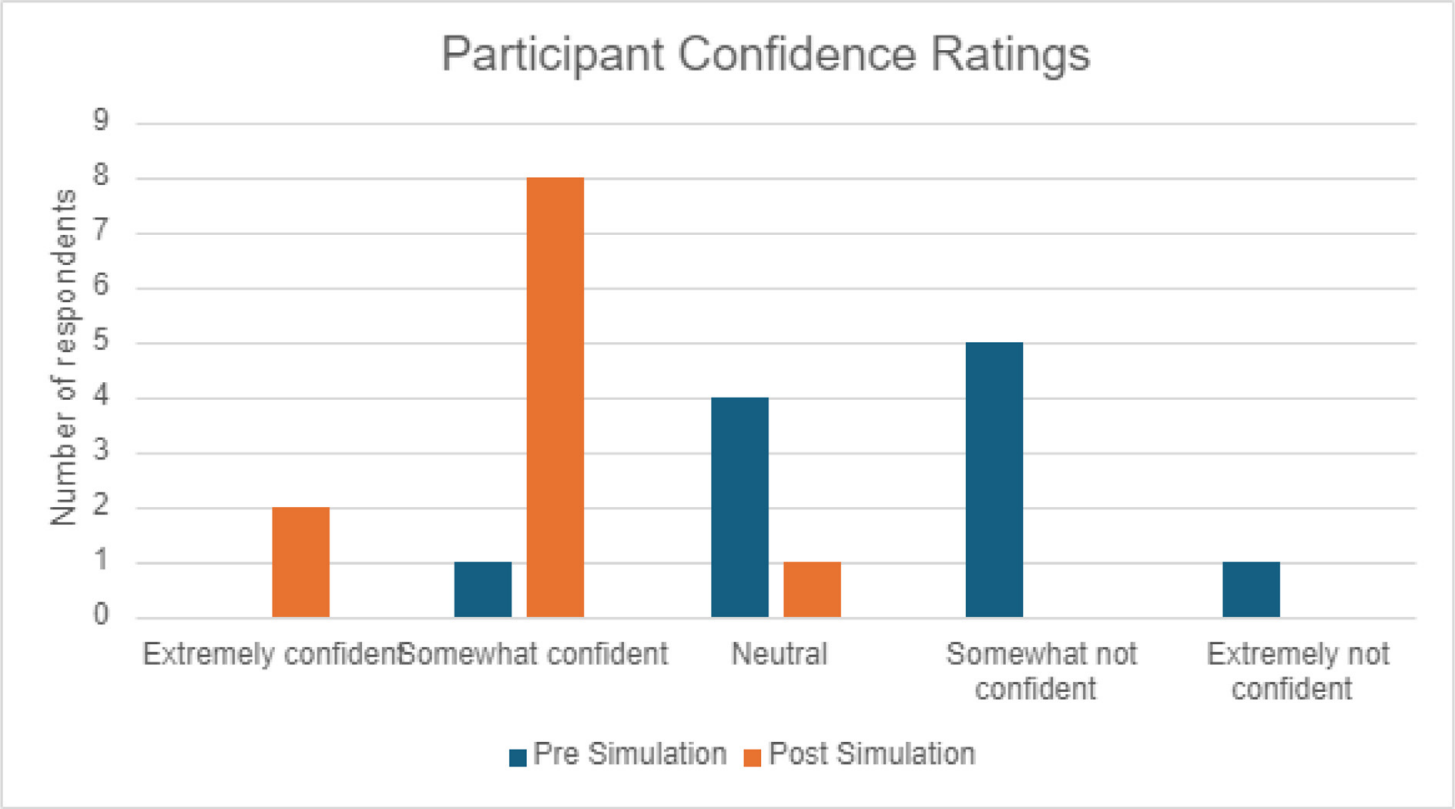
Participants’ self-rated confidence regarding their abilities to work as a medical doctor in Withybush, measured pre- and post-emergency ABCDE simulation.

Participants showed an increase in self-reported confidence following the emergency simulation, with only 9% (n=1) rating themselves extremely confident or somewhat confident in their abilities to work as a medical doctor in Withybush rising to 91% (n=10) following the simulation (Fig. 1).

Participants rated their overall experience of the ABCDE simulation as 9.82/10, commenting that it had been ‘really helpful’.

### On-call simulation session

**Fig. 2:**
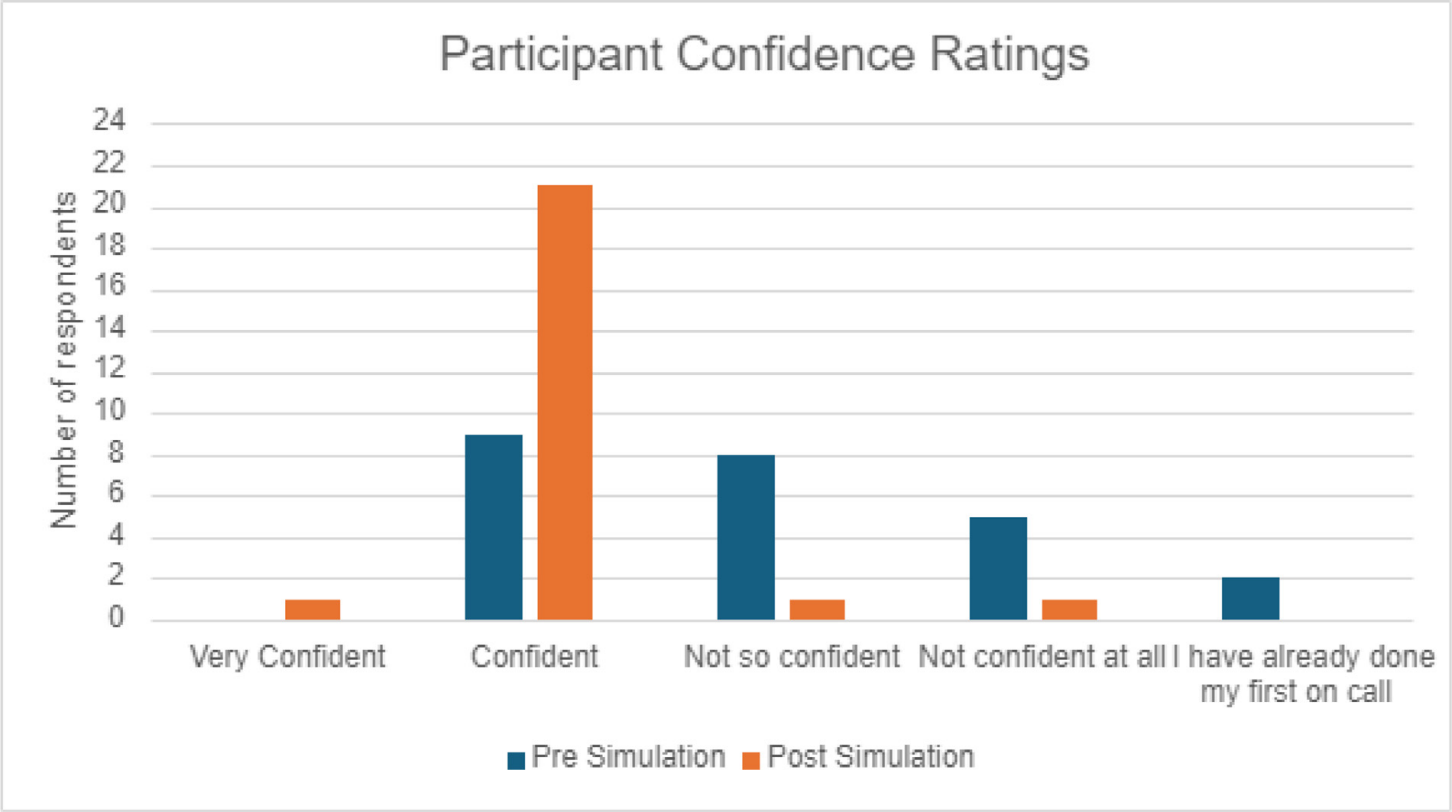
Participants self-rated confidence regarding their abilities while on-call, measured pre and post on-call simulation.

Following the simulated on-call, there was an increase in self-reported confidence among the IMGs (n=24), with only 38% (n=9) rating themselves confident or very confident about their first on-call before the simulation, rising to 92% (n=22) following the simulation (Fig. 2).

Those who had already completed an on-call (n=2) reported that their real life on-call experience had been ‘a bit chaotic’ and ‘so overwhelming’, recognising that the experience is a ‘steep learning curve’. Regarding the on-call simulation, these doctors reported that they ‘wish I had done this session much earlier’, which led to the creation of the monthly rolling induction roster.

Feedback demonstrated that our attempts to introduce local specific paperwork and culture, such as Welsh names and addresses, was helpful, and this facilitated ‘a very good experience and chance to see things in real and understand how things work here.’

### Educational supervisors

All educational supervisors who completed the survey (n=4) strongly agreed that: ‘The feedback provided enhanced my capacity to effectively support the doctor's professional development’. Furthermore, 100% of participating supervisors strongly agreed or agreed that the feedback improved the doctors’ ability to integrate into Withybush and work safely.

One participant commented that ‘The feedback provided is incredibly useful and detailed. It is highly transferable to the ward environment and provides a great platform in which to encourage and specifically focus on a particular area of development for each individual. I think the fact that the feedback is not generic and is highly specific, offers workable goals is what makes this feedback quite unique and very useful.’

### Limitations

As the programme was dynamically developed over the period of 13 months, numbers of participants providing feedback for the four sessions varied between activities and feedback was lacking in some areas.

Due to service needs, the shadowing period varied between IMGs in their clinical work, meaning that some doctors did not have the agreed 4-week period before entering on-call commitments and did not complete this induction until they had already begun working.

The number of educational supervisor participants who completed the survey of the feedback provided was low. Thus, it is difficult to extrapolate results from this survey. However, detailed free text qualitative feedback from the survey was extremely useful and allowed development of established feedback mechanisms.

This valuable programme involved an extremely committed multidisciplinary team who dedicated significant time and resource to its design and delivery, including provision for IMGs to be away from their clinical duties, which may not be possible in all settings.

## Conclusion and next steps

This induction programme has promoted the integration of IMG doctors into our workforce by providing them with early support, feedback and location-specific skills. By building confidence and providing the opportunities to apply knowledge in a specific context, IMGs are better prepared for clinical work within the NHS. This also shows the importance of enabling educational supervisors to work with the educational faculty, utilising the IMG doctors’ written feedback to support the doctor to grow in their role.

This induction was positively regarded by the Royal College of Physicians during their hospital site visit, where the team were invited to share this good practice at the RCP College Tutor Meeting on a national platform.

Future work in this area would aim to assess the impact of the programme on retention of IMG doctors long term, as well as their professional progress at ARCP-style reviews, and success of their applications to training. Taking the project further, we could incorporate and address themes of wellbeing and mental health and could encompass an introduction on how IMGs begin to build a portfolio in the NHS, as well as reviewing doctors ‘ARCP review’ outcomes to identify areas for development in this established IMG induction programme.

A recognised limitation in the current induction was educational supervisor engagement, therefore future work could target how to effectively allocate supervisors to IMG doctors to improve training and promote engagement.

## CRediT authorship contribution statement

**Julia McLaughlin:** Writing – review & editing, Writing – original draft, Resources, Project administration, Methodology, Investigation, Formal analysis, Data curation, Conceptualization. **Katherine Baker:** Writing – review & editing, Resources, Project administration, Methodology, Investigation, Conceptualization. **Tracy Sandell:** Resources, Project administration. **Shirly Mathias:** Project administration. **Subramaniam Nagasayi:** Writing – review & editing, Methodology, Conceptualization. **Ilona Schmidt:** Supervision. **Christopher James:** Supervision, Methodology, Investigation, Conceptualization.

## Declaration of competing interest

The authors declare that they have no known competing financial interests or personal relationships that could have appeared to influence the work reported in this paper.

## References

[1] M. Lee D, Nichols T (2014). Physician recruitment and retention in rural and underserved areas. Int J Health Care Qual Assur.

[2] Valero-Sanchez I, McKimm J, Green R (2017). A helping hand for international medical graduates. BMJ.

[3] Council GM. Register data summary. 2024.

[4] Lane J, Shrotri N, Somani BK (2024). Challenges and expectations of international medical graduates moving to the UK: an online survey. Scott Med J.

[5] Council GM (2022).

[6] Rasquinha M (2022). Difficulties and educational challenges faced by international medical graduates in trust grade roles in the UK. Br J Hosp Med.

[7] Kehoe A, McLachlan J, Metcalf J, Forrest S, Carter M, Illing J (2016). Supporting international medical graduates’ transition to their host-country: realist synthesis. Med Educ.

[8] Hashim A (2017). Educational challenges faced by international medical graduates in the UK. Adv Med Educ Pract.

[9] Morrow G, Rothwell C, Burford B, Illing J (2013). Cultural dimensions in the transition of overseas medical graduates to the UK workplace. Med Teach.

[10] Gambhir N, Gangadharan A, Pope L (2024). Knowing me, knowing you: evaluation of the impact of trainer involvement at an enhanced induction programme for International Medical Graduates (IMGs). Educ Prim Care.

